# Chondrocyte death involvement in osteoarthritis

**DOI:** 10.1007/s00441-022-03639-4

**Published:** 2022-05-26

**Authors:** S. Salucci, E. Falcieri, M. Battistelli

**Affiliations:** 1 Department of Biomolecular Sciences (DiSB), Urbino University Carlo Bo, Via Cà le Suore, 2, Campus Scientifico Enrico Mattei, 61029 Urbino (PU), Italy; 2 Cellular Signalling Laboratory, Department of Biomedical and NeuroMotor Sciences (DIBINEM), University of Bologna, 40126 Bologna, Italy

**Keywords:** Micromass, Chondrocyte, Apoptosis, Necrosis, Osteoarthritis

## Abstract

Chondrocyte apoptosis is known to contribute to articular cartilage damage in osteoarthritis and is correlated to a number of cartilage disorders. Micromass cultures represent a convenient means for studying chondrocyte biology, and, in particular, their death. In this review, we focused the different kinds of chondrocyte death through a comparison between data reported in the literature. Chondrocytes show necrotic features and, occasionally, also apoptotic features, but usually undergo a new form of cell death called Chondroptosis, which occurs in a non-classical manner. Chondroptosis has some features in common with classical apoptosis, such as cell shrinkage, chromatin condensation, and involvement, not always, of caspases. The most crucial peculiarity of chondroptosis relates to the ultimate elimination of cellular remnants. Independent of phagocytosis, chondroptosis may serve to eliminate cells without inflammation in situations in which phagocytosis would be difficult. This particular death mechanism is probably due to the unusual condition chondrocytes both in vivo and in micromass culture. This review highlights on the morpho-fuctional alterations of articular cartilage and focus attention on various types of chondrocyte death involved in this degeneration. The death features have been detailed and discussed through in vitro studies based on tridimensional chondrocyte culture (micromasses culture). The study of this particular mechanism of cartilage death and the characterization of different biological and biochemical underlying mechanisms can lead to the identification of new potentially therapeutic targets in various joint diseases.

## Osteoarthritis

Osteoarthritis (OA) is one of the most studied joint pathologies which involves articular cartilage alterations (Zheng et al. 2021; Sacitharan 2019; Eitner et al. 2017; Zhang et al. 2016). In particular, cartilage damage and chondrocyte loss occur with aging (Chen et al. 2017; Jørgensen et al. 2017) and after joint injury (Loeser 2009; Grogan and D'Lima 2010; Cao et al. 2013). Moreover, inflammatory diseases, such as rheumatoid arthritis, affect approximately 1% of the population and represent another significant contributor to the premature failure of articular cartilage (Korb et al. 2009). In the USA, cartilage diseases affect more than 4 million persons which resort to knee-replacement surgery (Schumacher and Pieringer 2017; Claessen et al. 2017; Yu et al. 2016).

Joint injuries are very common among young athletes (e.g., anterior cruciate ligament or meniscal tears) and can frequently favour the onset of early osteoarthritis among those affected (Salzmann et al. 2017; Gouttebarge et al. 2014; Kuijt et al. 2012).

OA is one of the most common diseases affecting the synovial joints (approximately 40% in men and 47% in women for knee and hip OA) (Johnson and Hunter 2014). OA is characterized by slowly progressive degeneration of joint cartilage components, pain, stiffness, and functional limitation or loss of joint. It appears in the adult population with the highest economic impact, generally affecting both sexes (Johnson et al. 2008; Olivotto et al. 2007; Thomas et al. 2011). This disease leads to a significant reduction in the quality of life (Roos 2005; Vo set al. 2013; Neogi and Zhang 2013; Johnson and Hunter 2014) of millions of human beings, being a major cause of disability and therefore representing a huge health and socioeconomic burden (Cross et al. 2014). OA is a joint disease with different aetiologies and multifaceted risk factors. Several studies show an OA increase with age: usually, it develops after the age of 45 and the majority of people above 65 show a certain radiographic evidence of OA in at least one or more joints.

Therefore, advancing age is a major risk factor, and given the aging population in the developed world, the onset of OA is projected to increase in the coming decades (Ackerman et al. 2017; Jinks et al. 2004).

Gender is also recognized as a contributing factor, with the female population generally being at a higher risk of developing OA. Females are seen to develop more severe knee and hand OA compared to their male counterparts, especially when ≥ 55 years old (Srikanth et al. 2005). In fact, it has been reported that hormones may play a role in the increased incidence of OA in females, due to postmenopausal decrease in estrogen levels. Other OA risk factors are genetic predisposition, obesity, physical activity, previous joint injury, joint malalignment, and abnormal joint shape (McCulloch et al. 2017).

The analysis of the modifications of the articular cartilage is necessary because the integrity of the articular structures is fundamental in preserving the functionality and stability of the joint. Traumatic and degenerative joint changes are often associated with a high risk of OA, but the risk is greater after degenerative tears. The degeneration of the joint structures, which can also occur in the young and the elderly, is defined as structural and functional insufficiency of the tissue and is caused by various factors such as repetitive trauma and joint misalignments (Battistelli et al. 2019). Moreover, cell death can contribute to articular cartilage damage and it is correlated to a number of cartilage disorders in OA pathogenesis (Johnson et al. 2008; Olivotto et al. 2007; Thomas et al. 2011).

## Articular cartilage in healthy and OA patients

### Healthy cartilage

Healthy articular cartilage is a particular hyaline cartilage, 2 to 4 mm thick, which does not have blood or lymphatic vessels and nerves. Articolar cartilage contains a small number of cells named chondrocytes, that are immersed in a dense extracellular matrix (ECM). The matrix consist of water, collagen fibres, and proteoglycans and contains a smaller amount of other different proteins. The different components of the cartilage have a characteristic distribution in a various articular cartilage layers: the superficial area, the median area, the radial layer, and the calcified areas.

The superficial zone is very thin (tangential layer) and protects the deeper layers from shear stresses; it constitutes approximately 10 to 20% of the articular cartilage thickness. The collagen fibres of this area (mainly type II and IX collagen) are tightly packed and aligned parallel to the joint surface (Martin and Buckwalter 2001).

**Fig. 1:**
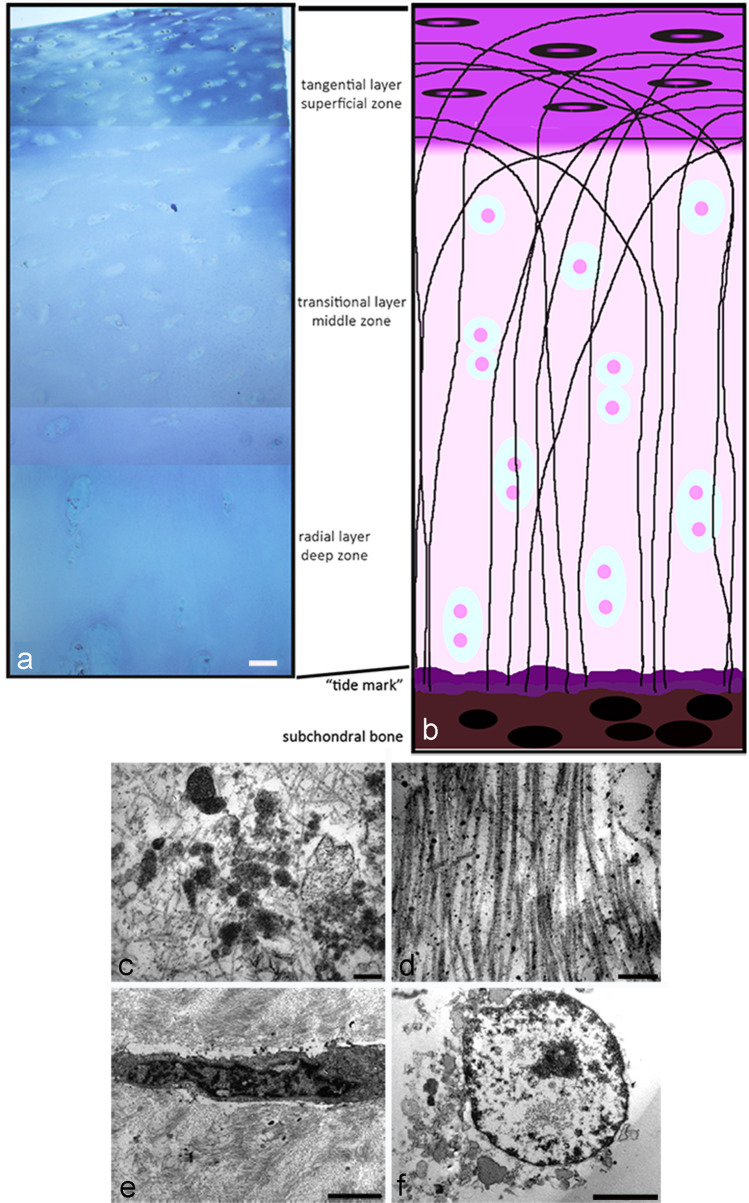
**A** Normal cartilage section is displayed after toluidine blu staining; this is a cartilage of young patient. **B** Schematic representation of articular cartilage. **C**, **D**, **E**, **F** TEM of osteoarthritis cartilage; image of small fragment of cartilage derived from old patient with articular cartilage degeneration

In the superficial layer, we can observe a high number of flattened chondrocytes, which are in contact with the synovial fluid, responsible for most of the mechanical properties of the cartilage, allowing it to resist the forces of traction and compression imposed by the diarthrotic joint. (Fig. 1A, B). Figure 1A, B are respectively an electron microscope image of control cartilage (A) and a figure of the same cartilage where we represent different layer (B).

The middle zone (transitional layer) provides an anatomic and functional bridge between the superficial and deep layers, and represents the first line of resistance to compression forces. This transitional zone is the most voluminous and represents about 60% of the total cartilage volume; it contains proteoglycans and thicker collagen fibrils organized obliquely, while the chondrocytes are spherical and at low density (Fig. 1A, B). The deep zone (radial layer) is responsible for providing the greatest resistance to compressive forces. The ECM shows a highest proteoglycan content and the lowest water concentration with the largest diameter collagen fibrils which are arranged perpendicular to the articular surface. The chondrocytes are disposed in columnar formation, parallel to the collagen fibres and perpendicular to the joint line. The deep zone represents approximately 30% of articular cartilage volume (Fig. 1A, B).

The “tide mark” separates the deep zone from the calcified cartilage. The deep zone, thanks to the high proteoglycan content and to collagen arrangement (it is perpendicular to articular cartilage surface), is able to resist to compressive forces,.

The calcified layer plays an integral role in securing the cartilage to bone, by anchoring the collagen fibrils of the deep zone to subchondral bone. In this zone, the cell population is scarce and chondrocytes are hypertrophic (Fox et al. 2009).

In each layer, the chondrocytes are the only cell type present. In healthy adult cartilage, chondrocytes synthesize matrix components very slowly and maintain a tight balance between synthesis and degradation of the ECM, including type II collagen and proteoglycan turn-over (Battistelli et al. 2019).

### OA cartilage

When alterations or trauma occur, chondrocytes proliferate and increase synthesis of the ECM components in an attempt to repair the defects. Therefore, they have a fundamental role in articular cartilage disorders including AO disease where morphological and functional changes of these cells seem responsible for the development of this pathology.

A variety of anabolic cytokines and growth factors stimulate chondrocytes to maintain matrix turnover with a delicate balance between synthesis and degradation (Sandell et al. 2001) which appears altered in OA.

In particular, in osteoarthritic states, due to excessive production of inflammatory cytokines and enzymes that degrade the matrix, tissue homeostasis is deeply modified and the ECM new synthesis is reduced. As a consequence, ECM loss and chondrocyte death are the central features in articular cartilage degeneration, during osteoarthritis pathogenesis (Pascarelli et al. 2015).

Over the last two decades, increasing evidence showed association between cartilage degradation and chondrocyte death in OA and rheumatologic diseases (Bertrand et al. 2010).

Chondrocytes are specialized cells of articular cartilage that are integrated in the intercellular substance; they are scattered in cartilage cavities, also called lacunae. Lacunae can contain one or more chondrocytes. They are rounding or slowly elongated 10–20 µm cells. Each chondrocyte can be considered as a functional single unit with a fundamental role in the new synthesis and maintenance of the ECM in its proximity.

Various attempts have been carried out to obtain in vitro chondrocyte culture. The principal limitation is due to the peculiar biology of these cells, which, in vivo, reside in a tridimensional environment, surrounded by a well-defined ECM. In fact, outside their native environment and when cultured in monolayer, the dedifferentiate cells and, as a consequence, the production of ECM components, decreases (Li et al. 2012; Mazzetti et al 2004; Kitamura 2004; Schnabel et al. 2002; Wiseman et al. 2004).

However, an in vitro system is required to investigate and highlight on chondrocyte behaviour in normal and pathological conditions.

Osteoarthritis cartilage is fragmented; this is a morphological evaluation of Oa cartilage of old patient with knee joint cartilage degeneration. Observation of large pieces of OA cartilage is very difficult; we have only small fragment (Fig. 1C, D, E, F). Its ultrastructure is degenerated, we can observe the matrix calcifications (C), and the collagen fibres, that are poorly organized, are thin and without the typical banding (E).

The cells have an apoptotic (E) and sometimes necrotic morphology (F). Therefore, three-dimensional culture systems, such as hydrogels including agarose, alginate, Matrigel, and collagen type I hydrogels as cell substrates (Miao et al. 2017), are currently used to obtain the chondrocytes original phenotype. In particular, this review describes a tridimensional culture method which utilizes a high-density mobile system to induce chondrogenesis: micromasses culture. It represents an optimal system for the chondrocyte growth and allows a diffuse viability of chondrocytes that recover differentiated phenotype and tridimensional interactions with collagen 2 and ECM proteins (Battistelli et al. 2005).

## Micromass chondrocytes

For chondrogenesis studies, micromass culture appears a convenient system since it provides a three-dimensional environment where cell interactions are similar to those observed during embryonic development. To obtain micromass model, cartilage portions are processed with a sequential enzymatic digestion (Pulsatelli et al. 1999; Battistelli et al. 2005), and ECM-freed chondrocytes are maintained in culture (D-MEM, 10% FCS). Chondrocytes (500,000) at first passage are pelleted (by centrifugation for 20 min) and then differentiated in the same medium to obtain an in vitro cartilage model (Pulsatelli et al. 1999). Chondrocyte behaviour in micromass culture has been described through morphological analyses with original images obtained at both light and transmission electron microscopes. Control and treated samples have been processed for ultrastructural analyses as detailed in previous papers (Battistelli et al. 2019; Battistelli et al. 2014).

**Fig. 2:**
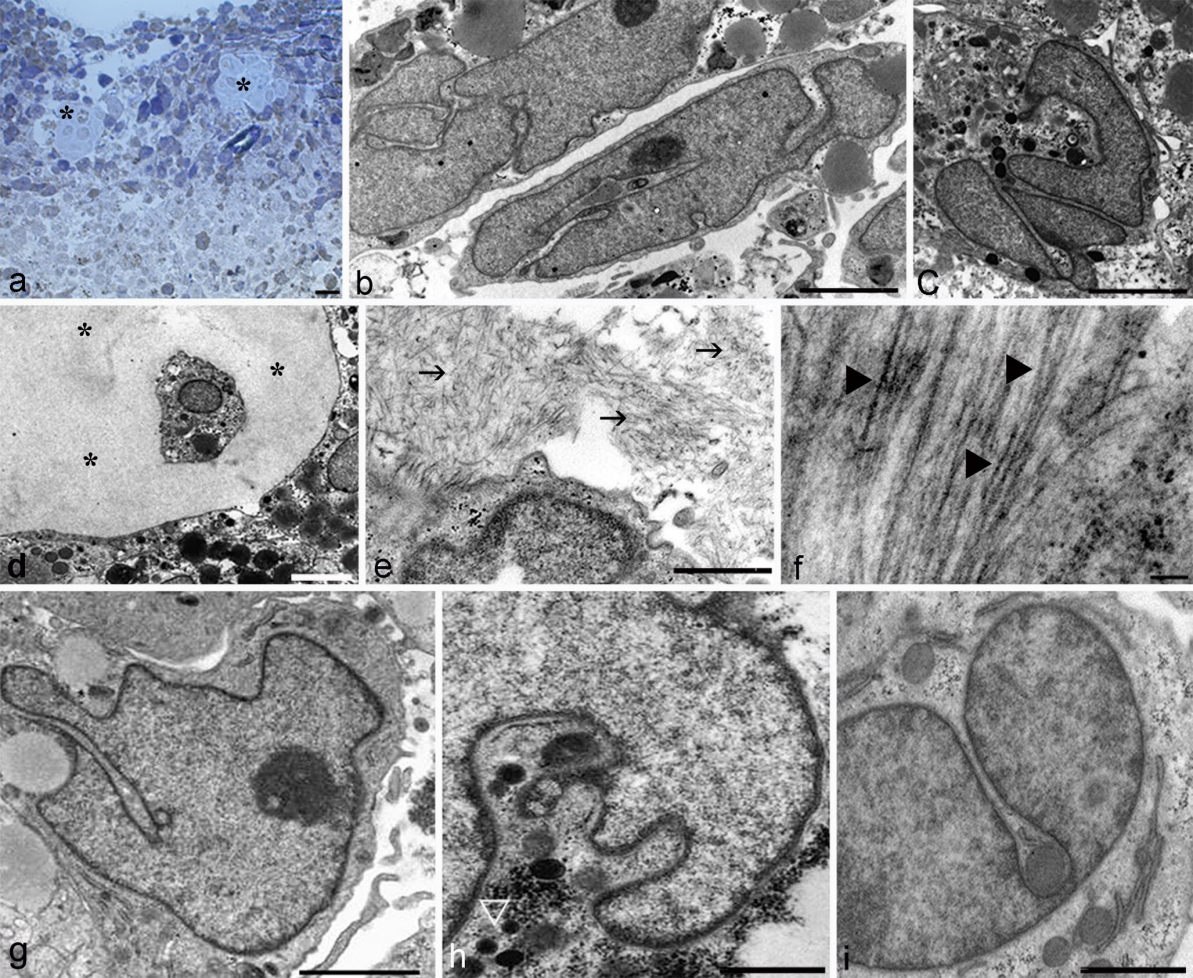
Optical microscope (**A**) and TEM of control chondrocyte in micromass (**B**–**I**). Micromass appears as a sphere of 1 mm diameter (**A**). Flattened chondrocyte at micromass periphery and ovoidalin in the inner micromass (**B)** can be observed. Occasionally, they are scattered in cartilage cavities, also called lacunae (**A**, **D**) (*). The progressive ECM assembling appears mainly distributed in close cell proximity (**E**). Chondrocyte are surrounded by a complex matrix, formed by proteoglycans (**E**, →) and collagen fibres (**F**, ►). Diffuse nuclear chromatin (**B**, **C**, **F**, **G**, **H**, **I**) and well preserved organelle (**G**, **H**, **I**) can be observed. **A** Bar 10 µm; **B**, **C**, **E**, **F**, **G**, **H**, **I**, Bar 2 µm; **D** Bar 5 µm

At light microscope, micromass appears as a spheres of 1 mm diameter (Fig. 2A) with chondrocytes widely comparable with those of middle layer of the in vivocartilage (Battistelli et al. 2005).

Ultrastructural analyses show chondrocytes slightly flattened at micromass periphery (Fig. 2B) and ovoidal in the inner micromass (Fig. 2C). Occasionally, they are scattered in cartilage cavities, also called lacunae (Fig. 2A, D) (*).

Chondrocyte micromasses allow to obtain a ECM similar to that of native cartilage; in fact, it appears mainly distributed in close cell proximity (Fig. 2E).

Therefore, normal chondrocyte are surrounded by a complex matrix characterized by the presence of proteoglycans (→) and collagen fibres (►) in the intercellular space. These features indicate an active ECM production with proteoglycans – mostly in cell proximity – collagen fibrils, and matrix vesicles (Fig. 2E, F). Diffuse chromatin can be observed (Fig. 2B-I) as well as cytoplasmic organelles such as a Golgi bodies, smooth endoplasmic reticulum, rough endoplasmic reticulum rich in secretory material, and preserved mitochondria (Fig. 2G-I). Large glycogen masses and isolated granules, scattered throughout the cytoplasm, are further features characteristic of micromass chondrocytes (Fig. 2 H) (▷).

It is known that chondrocyte death is associated to ECM degradation by contributing to a chronic ECM remodelling process that characterizes AO. Cell death can lead to altered ECM structure and abnormal mechanical function. Therefore, to understand the type of death and the correlated pathways of articular cartilage degeneration allows to reach the strategies to prevent these alterations. In the following sections, chondrocyte death types (necrosis, apoptosis, and chondroptosis) have been described and discussed considering their behaviour when they have differentiated in micromass system and exposed to physical (hyperthermia and UVB) and chemical (staurosporine) triggers.

## Necrosis

Necrosis is a pathological and non-programmed form of cell death, caspase- and energy-independent (Zhang et al. 2018; Komori 2016; Martin and Henry 2013). Chondrocytes die by necrosis, in particular necroptosis after exposure to environmental stressors that cause chemical and mechanical injury, inflammation, or infection (Khoury et al. 2020). Furthermore, dead chondrocytes release intracellular components which lead to the generation of reactive oxygen species and inflammatory factors with consequent tissue damage (Zheng et al. 2020). The current literature suggests that necrosis and necroptosis in chondrocytes involved pro-inflammatory cytokines, including the TNF-α receptor system (Stolberg et al. 2020) which induce cartilage degradation. Necroptotic cell death seems to have a common origin with apoptosis, but a different behaviour occurs with promotes inflammation and disruption of the membrane integrity by causing the progression of degenerative disease.

**Fig. 3:**
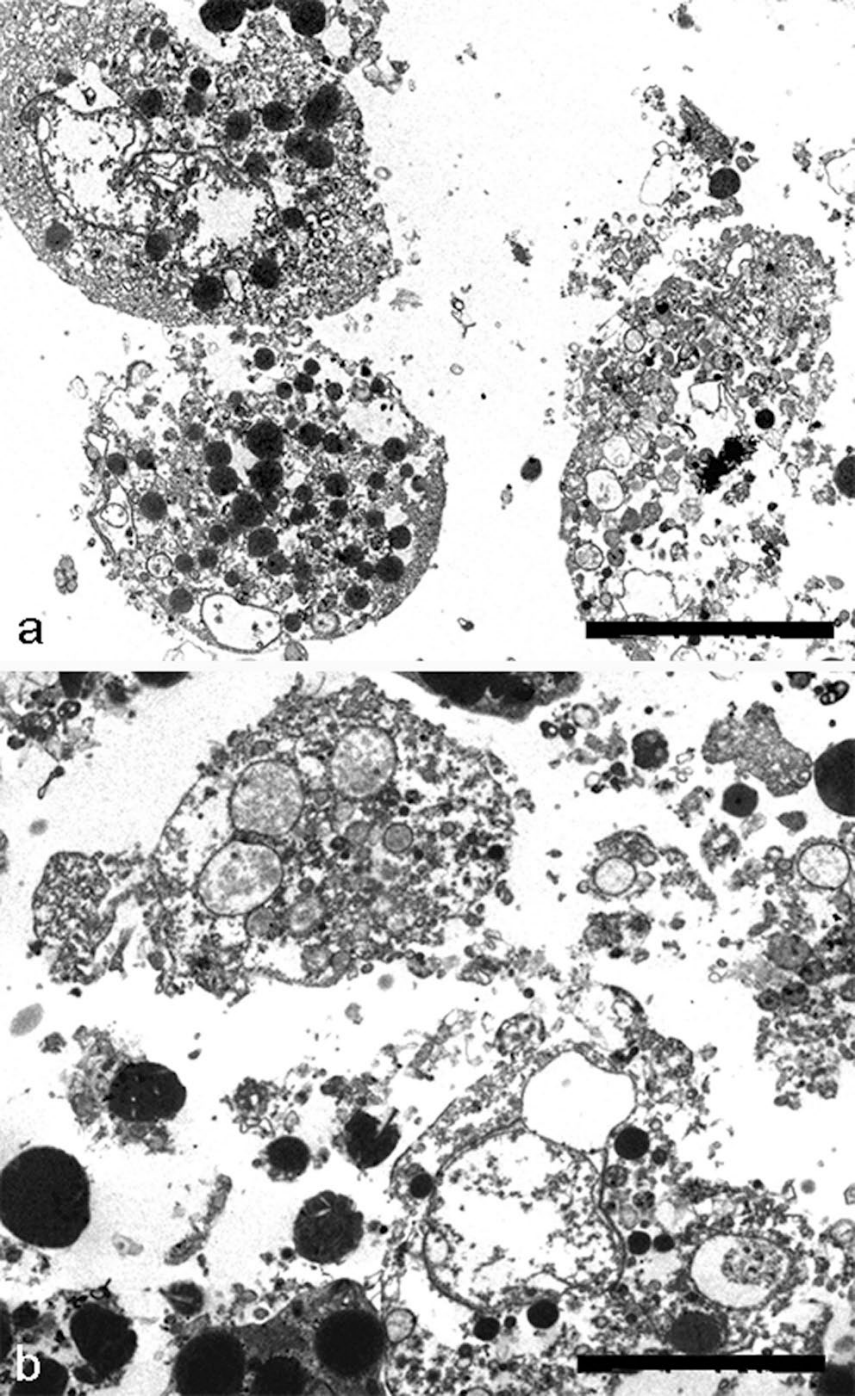
TEM of chondrocyte necrosis. The plasma membrane integrity is not maintained in necrotic chondrocytes (**A**, **B**). Cytoplasm appears emptied and total cell lysis can be observed (**A**, **B**). **A**, **B** Bar 2

The main difference between apoptotic and necrotic cell death is that the latter is always accompanied by inflammatory reaction (Vanden Berghe et al. 2013) in response to accumulation of cytoplasmic contents in intercellular regions, due to the loss of cell membrane integrity. Also, chondrocytes show necrotic features even if without phagocytosis it is difficult distinguish true necrosis from secondary necrotic cells which show the morphological features of primary necrosis (Fig. 3).

Necrotic death appears in chondrocytes cultured in micromass model (Charlier et al. 2016) in particular after prolonged exposure to UV radiation or after prolonged hyperthermic treatment, especially in aged micromasses and in the presence of complete loss of ECM (Fig. 3A, B). The plasma membrane integrity is not maintained, and they release their cellular contents, including lysosomal enzymes, into the extracellular space. They show characteristic necrotic changes, such as formation of cytoplasmic vacuoles. Cytosol and organellar components appear emptied, with general cell lysis (Fig. 3A, B).

## Apoptosis

Apoptosis, or programmed cell death, was first described by Kerr et al. (1972). This term was used to describe a cell death mechanism that plays a crucial role in maintaining the homeostasis of various tissues. Its dysregulation leads to pathological states, such as cancer, developmental anomalies, and degenerative diseases.

After tissue injury, a variety of cells, including neutrophils, macrophages, and lymphocytes, are accumulated in the injury site to repair the damage, and after the healing process, the surplus of accumulated cells is eliminated by programmed cell death to prevent excessive inflammation (Greenhalgh 1998; Aigner et al. 2004).

Apoptosis is a highly regulated pathway that involves specific sets of intracellular signals and genes. There are two classical ways for apoptosis, namely, the death receptor pathway and the mitochondrial one (Salucci et al. 2017). Both pathways for their execution require different enzymes called caspases, that cleave specifically at aspartate residue. Specific death ligands such as TNFα and Fas activated the death receptor pathway. While the mitochondrial pathway is initiated by external stimuli which modify mitochondrial membrane permeability by favouring the release of pro-apoptotic proteins.

Morphological apoptosis is characterized in the early stages by a progressive margination and clustering of dense chromatin, nuclear fragmentation, membrane blebbing, cell shrinkage, and a relatively good preservation of the cytoplasmic organelles.

During chromatin margination, nuclear pore redistribution around the nuclear envelope can be observed. Pores appear absent in the sites of the envelope corresponding to dense chromatin, while they appear clustered close to diffuse chromatin areas.

Late apoptotic cells are characterized by deep cytoplasmic rearrangements and micronuclei. Apoptotic bodies display micronuclei, sometimes surrounded by a double envelope, and nucleolar residues scattered throughout the cytoplasm.

A characteristic vacuolization of the cytoplasm, in the presence of a good preservation of the plasma membrane and organelles, is a remarkable feature of this phase.

**Fig. 4:**
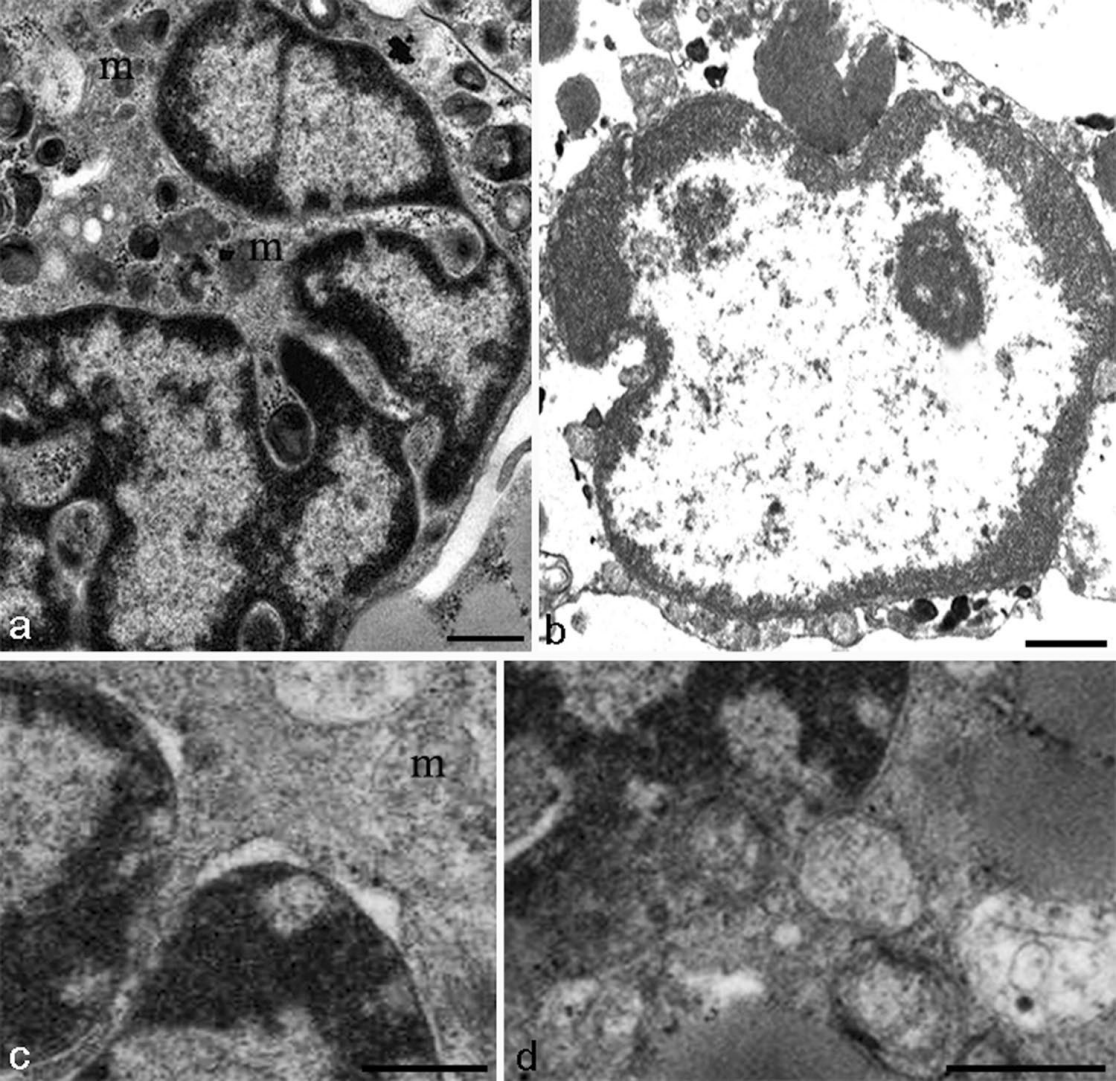
TEM of chondrocyte apoptosis. Nucleus appears lobed (**A**) and presents condensed chromatin, localized at the nuclear periphery (**A**, **B**). The outer nuclear membrane appears occasionally detached at condensed chromatin level (**C**). Some nuclear pores appear close to diffuse chromatin (**C**, **D**). Rounding and swollen mitochondria can be revealed (**A**, **C**) (m). True apoptotic bodies are only rarely found (**E**). **A**, **C**, **D** Bar 1 µm. **B** Bar 2 µm

These morphological patterns occasionally appear, in chondrocytes after UVB exposure or staurosporine treatment, known apoptotic trigger. In particular, chromatin condensation (Fig. 4A, B), pore translocation, and clustering, typical features of apoptotic nuclei (Fig. 4C, D), can be observed.

The nucleus is lobed (Fig. 4A), with condensed chromatin, localized at the nuclear periphery (Fig. 4A, B). The outer nuclear membrane sometimes appears detached at condensed chromatin level (Fig. 4C), and nuclear pores can be revealed close to diffuse chromatin (Fig. 4C, D). In the cytoplasm, rounding and swollen mitochondria are present (Fig. 4A, C) (m).

Apoptotic bodies, elsewhere a common marker of apoptosis, are rarely found in this tissue (not shown), as demonstrated by extensive researchers for many years, for instance Zamli and Sharif (2011), Battistelli et al. (2014), Millucci et al. (2017), and Roach et al. (2004).

## Chondroptosis

This term proposed by Roach et al. (2004) describes a new form of cell death, which occurs in a non-classical manner as will be shown below, but in a way that seems typical for programmed chondrocytic cell death in vivo.

Chondroptosis is a peculiar type of cell death which affects cartilage. It can be found in osteoarthritis, rheumatic disease (Millucci et al. 2015), and after trauma (Sitte et al. 2009). In our in vitro model, chondrocyte death appears when micromass cultures are exposed to hyperthermia.

Hyperthermia (HT) has become the fifth strategy, after surgery, chemotherapy, radiation, and biological therapy in the fight against cancer (Salucci et al. 2015). It plays an important role in multidisciplinary treatments, representing a very promising method that will be seriously considered in the future. Hyperthermia, a powerful cell death inducer, has been demonstrated to induce chondroptosis in chondrocytes too (Battistelli et al. 2014). Hyperthermia-induced chondroptosis is characterized both by its distinctive ultrastructural patterns and by the occurrence of internucleosomal DNA cleavage in most cells (Battistelli et al. 2014). In addition, heat exposure is a powerful apoptotic inducer in a variety of cells, where it induces classical apoptotic changes and the known biochemical pathways, but the underlying mechanisms are only partially understood.

**Fig. 5:**
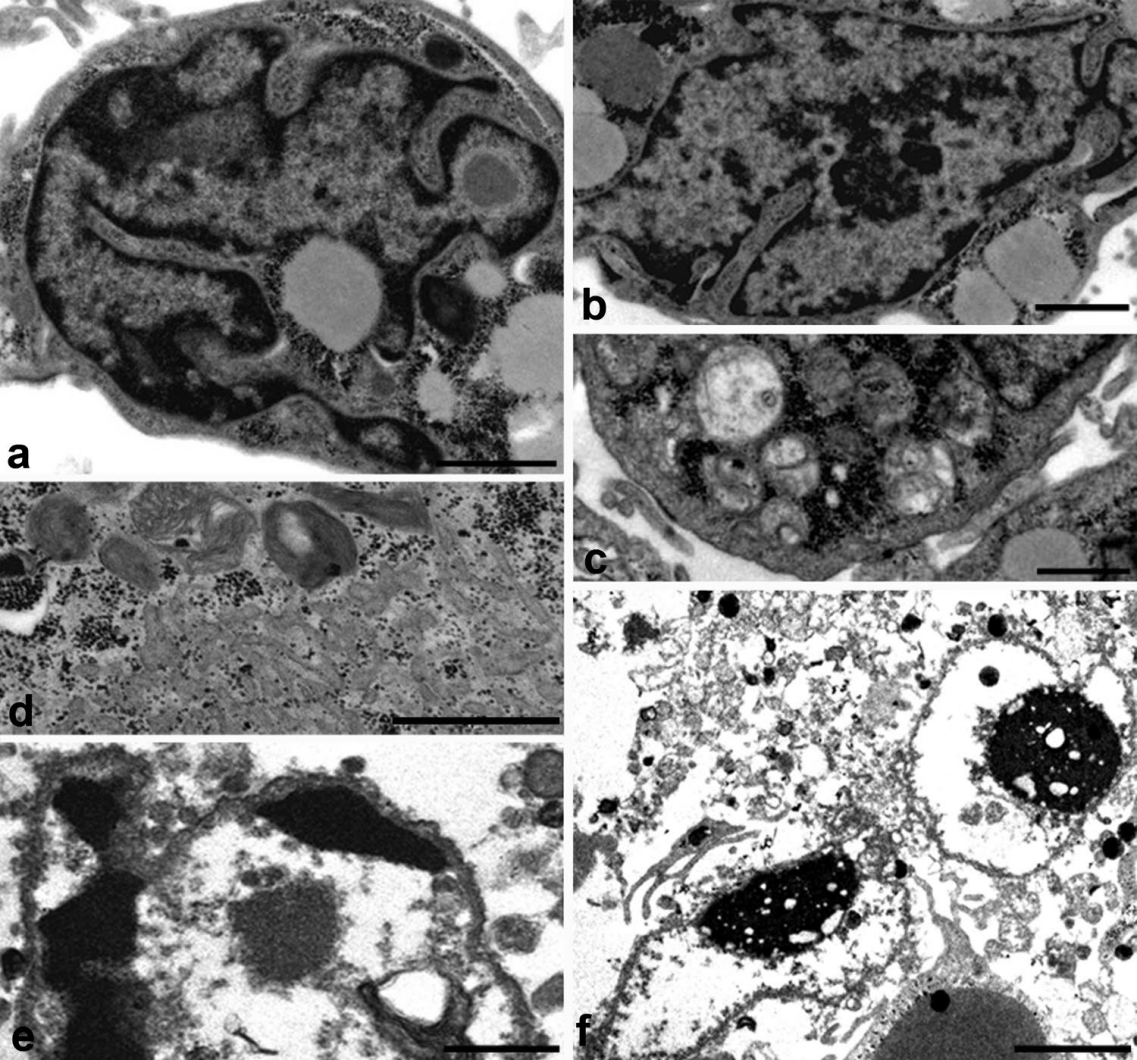
TEM of chondrocyte chondroptosis. Cellular shrinkage and chromatin condensation (**A**, **B**) can be observed. Chromatin is marginated into small patches spread throughout the nucleus (**A**, **B**). The presence of autophagic vacuoles (**C**) and rough endoplasmic reticulum (RER) appear increased (**D**). Nucleus, with very condensed chromatin, displays electron-dense agglomerates in close proximity of nuclear membrane (**D**, **E**)

Evidence is also accumulating that hyperthermia may act through mitochondrial mechanisms and reactive oxygen species accumulation. Cellular shrinkage and chromatin condensation are in common with classical apoptosis (Fig. 5A, B). However, in contrast to classical apoptosis, the chromatin is not marginated into large solid masses, but small patches of condensed chromatin are spread throughout the nucleus (Fig. 5A, B). Sometimes the nucleus has a very convoluted appearance, also described by Millucci et al. (2017). The features that most distinguish chondroptosis from classical apoptosis are also the frequent presence of autophagic vacuoles (Fig. 5C), responsible for the extrusion of cellular material into the extracellular space. Rough endoplasmic reticulum appears increased and very expanded (Fig. 5D). Nucleus displays electron-dense agglomerates in close prossimity of nuclear membrane (Fig. 5D, E). In agreement with Almonte-Becerril et al. (2010), different authors have observed that cell death of chondrocytes undergoes changes different from the classical apoptosis. Almonte-Becerill suggested that this type of death is a combination between the classical apoptosis and autophagy (Almonte-Becerril et al. 2010).

**Fig. 6:**
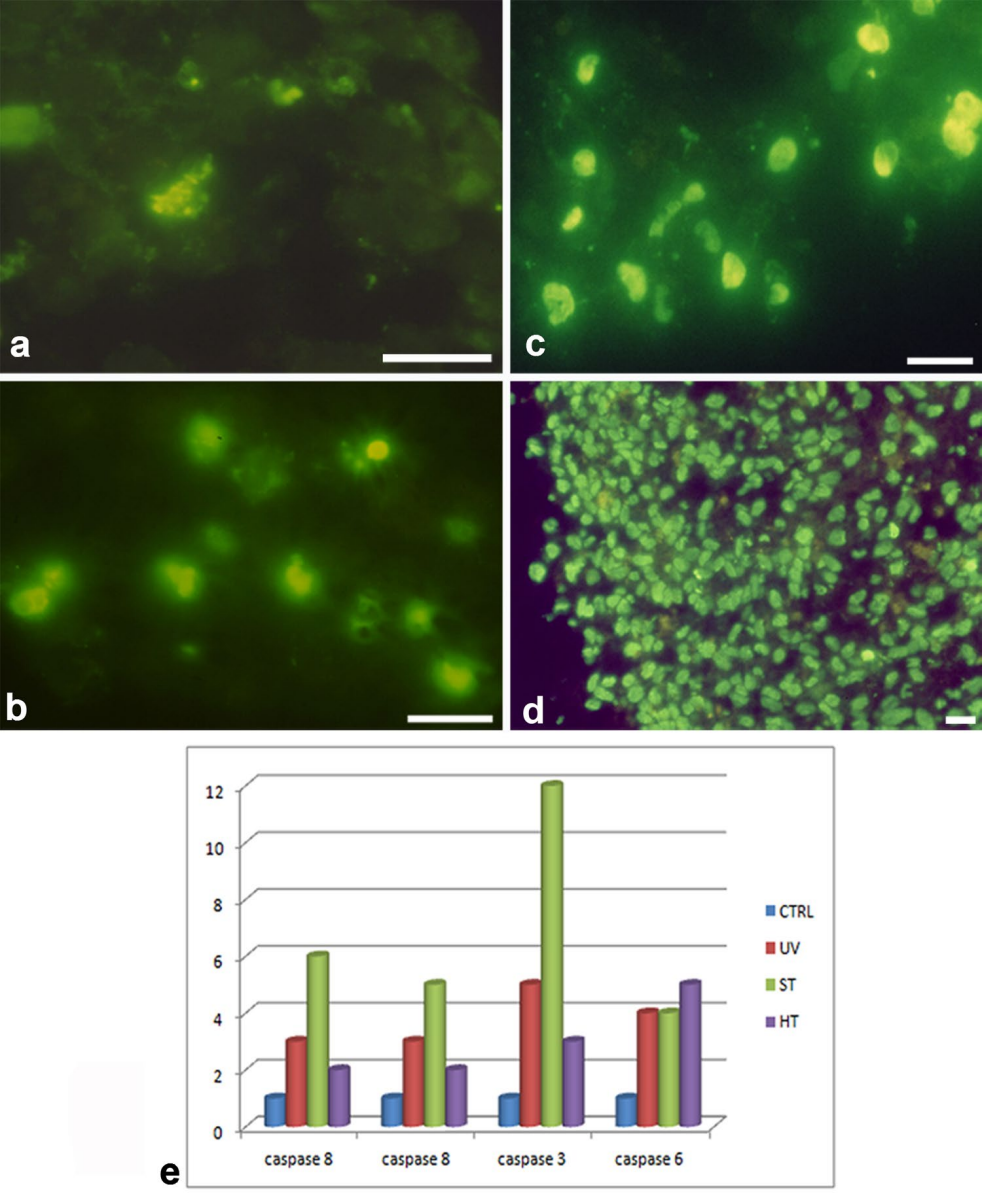
TUNEL reaction (**A**, **B**, **C**, **D**) and caspase activation (**E**). Control cells appear negatively stained (**A**) but after staurosporine (**B**), UVB (**C**), and hyperthermia (**D**) treatment positive cells can be observed. **E** Caspase-8, -9, -3, and -6 expression in control, staurosporine (ST), hyperthermia (HT), and UVB (UV) conditions. **A**, **B**, **C**, **D**, **E**, **F** Bar 1 µm

Some type of DNA cleavage can be evidentiated in chondroptosis, since the cells show a positive reaction with the TUNEL method (Fig. 6) (Charlier et al. 2016). Control cells appear negatively stained (Fig. 6A), but after staurosporine (Fig. 6B), UVB (Fig. 6C) and hyperthermia (Fig. 6D) treatment, positive cell nuclei can be observed.

To further characterize the type of programmed cell death occurring in chondrocyte micromasses, it is necessary, through molecular analyses, investigate the presence of DNA fragmentation and the caspase activation.

DNA electrophoresis on agarose gel shows the lack of the classical apoptotic ladder. In fact, DNA samples extracted from micromasses show a minimal, yet appreciable higher mobility, suggesting a relevant occurrence of high molecular weight DNA fragments in these samples (Cross et al. 2014). The presence of in situ DNA cleavage after TUNEL reaction has been demonstrated after apoptotic treatments with an elevated number of TUNEL positive nuclei, in particular, in aged micromasses. Jiang et al. showed that TUNEL staining positivity characterized chondrocytes in intervertebral disc degeneration, by confirming the presence of DNA fragmentation as hallmark of chondroptosis (Jiang et al. 2017).

In chondrocytes death, caspase behaviour appears a peculiar mechanism. Micromass lysates were tested with selective fluorogenic substrates to better characterize the type of caspase pathway activated by the different stimuli and to investigate the involvement of effector (–3 or –6) or initiator (–8 and –9) caspases. Chondroptosis induced by hypertermia (HT) is associated to a very modest activation of caspase–8 and –9, substantially no activation of caspase–3, while caspase–6 is activated (Fig. 6E) (Battistelli et al. 2014). Chondrocyte apoptosis is associated, whether staurosporine (ST) treatment or UVB exposure (UV), to caspase–8, –9, and –3 activation (Fig. 6E). Therefore, chondroptosis involves common molecular features with classical apoptosis, such as caspase involvement and DNA cleavage.

## Conclusions

This review discusses the different kinds of chondrocyte death through a comparison between data reported in the literature, highlighting on morphological features of cell death when chondrocytes differentiate in a micromass model.

**Fig. 7:**
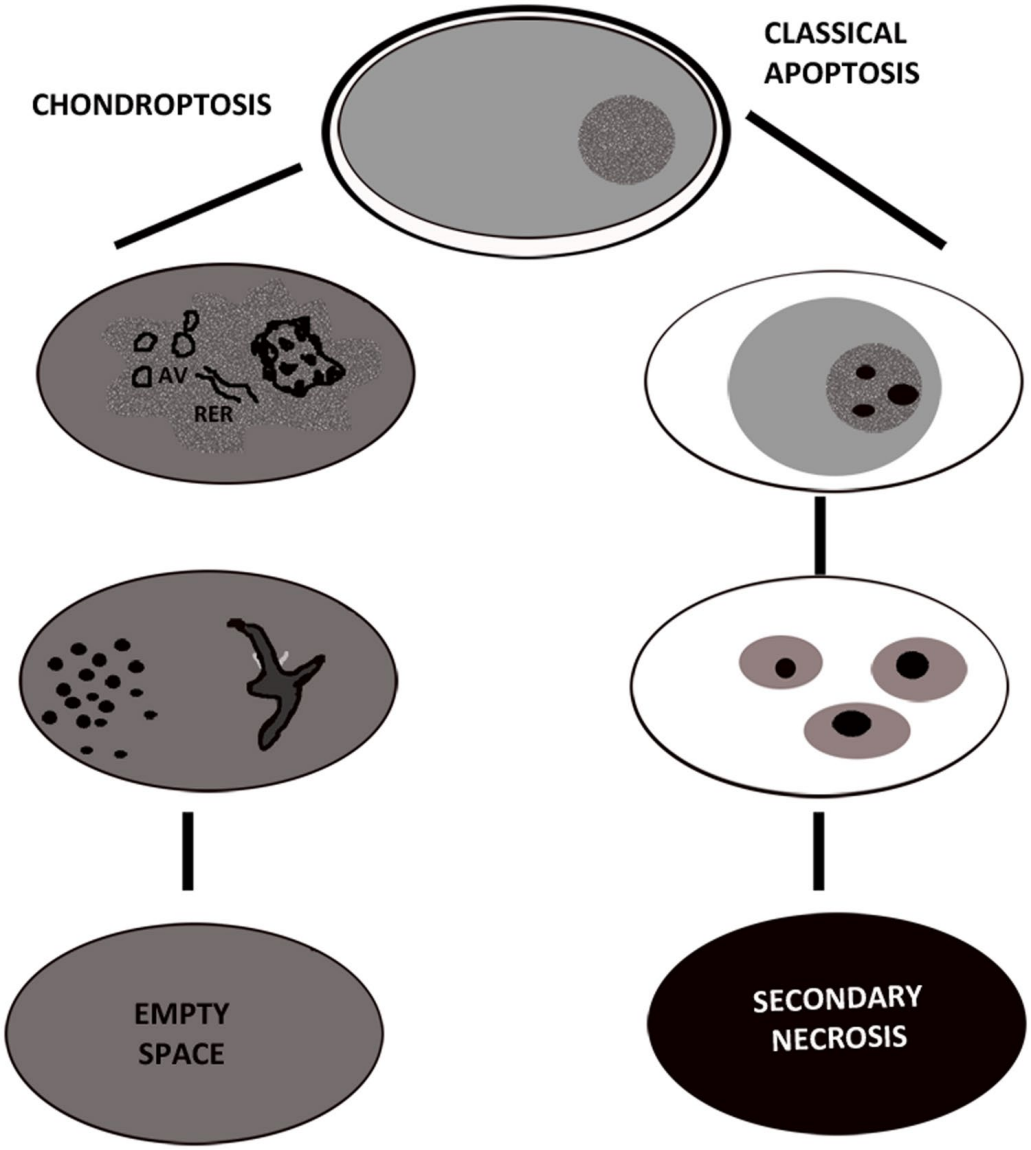
Illustration of the main and crucial differences between classical apoptosis and chondroptosis

The morphological and biochemical analyses reflect alternative mechanisms of cell elimination. The new term chondroptosis, proposed by Roach et al. was used to describe one type, probably the major type, of chondrocyte death. Chondroptosis has some features in common with classical apoptosis, such as cell shrinkage, chromatin condensation, and involvement, not always, of caspases. In particular, after hyperthermia, chondrocytes show caspase-6 activation even if the executive caspase, activated during classical apoptosis, appears down-regulated. The crucial and main differences between classical apoptosis and chondroptosis have been described in Fig. 7. The most crucial peculiarity of chondroptosis relates to the ultimate elimination of cellular remnants. Independent of phagocytosis, chondroptosis may serve to eliminate cells without inflammation in situations in which phagocytosis would be difficult. This particular death mechanism is probably due to the unusual condition chondrocytes both in vivo and in micromass culture. ECM could indeed influence the chondrocyte response, being chondrocyte closely surrounded by ECM more resistant to the stimuli. On the other hand, Karimi-Busheri et al. (2010) have previously reported, for other cell types, the DNA damage attenuation in high density culturing. Probably, the close connection between chondrocytes and ECM components, as well as cell density, exerts a sort of protective effect.

This behaviour has been observed in different models, such as muscle sincytia, which appear when stimulated, more resistant to a variety stimuli if compared to myoblastic mononucleated cells (Salucci et al. 2013).

To highlight on the mechanisms involved in cartilage death appears a fundamental research field to the identification and the development of new potentially therapeutic targets in various joint diseases. In this scenario, in vitro micromass culture of chondrocytes represents a promising model useful to mimic the developing cartilage in vivo, and to investigate the chondrocyte behaviour during cartilage degeneration.
